# Tweeting in the Time of Coronavirus: How Social Media Use and Academic Research Evolve during Times of Global Uncertainty

**DOI:** 10.1177/2056305120948258

**Published:** 2020-08-11

**Authors:** Neta Kligler-Vilenchik, Daniela Stoltenberg, Maya de Vries Kedem, Hadas Gur-Ze’ev, Annie Waldherr, Barbara Pfetsch

**Affiliations:** 1 The Hebrew University of Jerusalem, Israel; 2 WWU Munster, Germany; 3 Free University of Berlin, Germany

**Keywords:** crisis communication, COVID-19, Israel, survey research, Twitter

## Abstract

Our international research team was in the midst of a comparative study about the day-to-day experience of Twitter users in Berlin and Jerusalem through a series of daily short surveys, when our Jerusalem data were becoming increasingly “compromised” by the growing public concern, and tightening government measures, around the spread of the Coronavirus in Israel. During the two waves of our 10-day survey of salient Twitter users in Jerusalem (March 9–March 19, *N* = 34; March 23–April 2, *N* = 25), Israel shifted from 50 confirmed Coronavirus cases to over 6,800 and from relative routine to almost full stay-at-home orders. This essay presents two intersecting narratives. First, we consider the methodological challenges of adapting ongoing academic survey studies to changing conditions. We then offer a mixed-methods analysis of the experiences of our Twitter users and how they saw the Coronavirus crisis shaping their use of Twitter. The essay thus offers a unique methodological and empirical vantage point on how social media use—and academic research—evolve during times of global uncertainty.

The catchphrase “never let a crisis go to waste,” attributed to Obama-advisor Rahm Emanuel in the context of the 2008 financial crisis, has been made newly relevant in the context of COVID-19. Yet making the most of a crisis often means needing to act fast, and under conditions of uncertainty. Unlike political leaders, researchers may be less accustomed to fast-paced shifts of research agendas, but both may sometimes gain from a crisis. This essay presents two intersecting narratives: Our response to the evolving situation as a research team; and a glimpse of the resulting data we gained, reflecting shifting social media use during the initial escalation of the Coronavirus crisis in Israel.

## Doing Research under Changing Conditions—The Researchers’ Point of View

We are an international research team engaged in an ongoing study on the role of space and locality in Twitter communication, comparing two cities: Berlin and Jerusalem.^1^ In early 2020, we were in the midst of one of the peaks of the study—a three-language survey with salient Twitter users,^2^ using a ‘Mobile Experience Sampling Method’: a series of two short daily surveys asking about the last instance of Twitter usage, sent over 10 days via SMS. Our Berlin survey, completed in January–February 2020, was supposed to be compared with the Jerusalem survey, planned to occur in two waves during March–April 2020.

**Figure 1.**
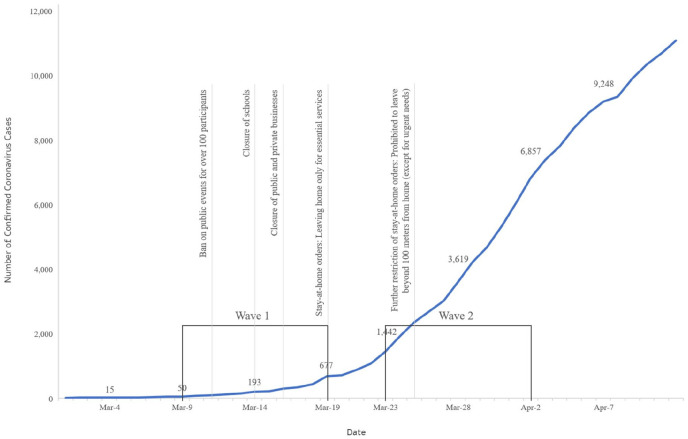
Confirmed Coronavirus cases and public measures per date and survey wave.

The Coronavirus seemed a distant event unrelated to our study. A first sign that it might interfere with our research came in early March—the second author, a German team member, had to cancel her planned research stay in Israel due to border closures. Despite this hiccup, we continued with the survey as planned. When the first wave began on March 9, Israel had 50 confirmed Coronavirus cases, and the public mood—while tense—was still one of relative routine. The 10 days of our first survey wave, however, proved to be dramatic in Israel. As Figure 1 illustrates, these days showed a fast pace of contagion, accompanied by increasingly tightening public measures, from school closures to stay-at-home orders.

Throughout this, our first wave respondents were filling out the survey twice daily. We quickly understood our data would be affected, making comparisons with the Berlin case moot.^3^ The key to “not letting the crisis go to waste” was acknowledging that our original, comparative research plan was no longer valid, and to consider what the new opportunities may be. Surprisingly, this direction emerged directly from the field. At the mid-point of our survey, a respondent informally shared that her tweeting habits were atypical: “I was just thinking that the Corona situation may taint your data—I keep answering that I’m tweeting from home at times when I would not normally be home. Just something to consider 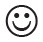.”

The respondent’s message provided the tipping point for us to see the Coronavirus as a research opportunity. On March 16, 3 days to the survey’s end, we decided to add a post-survey questionnaire examining how the situation affected respondents’ Twitter use. Over two hectic days, team members composed the post-survey questionnaire, translated it into three languages, implemented it into the survey system, and pilot-tested it. Since we were dealing with an unfolding crisis of an unknown nature, we were unsure standardized batteries of use motivations and intensity would capture relevant changes in our respondents’ social media use. We thus opted for a bottom-up approach and added the following item: “Please share with us in an open-ended manner how you feel the Coronavirus situation shaped your Tweeting habits over the past 10 days.” On March 19, our post-survey questionnaire was launched. The high response rate (89% of Wave 1 respondents) confirmed that our participants had much to share.

After the high-paced effort of preparing the Coronavirus survey, implementing it in the second wave of our survey (March 23–April 2) was smoother. As seen in Figure 1, during Wave 2 of our research, Israel remained in lock-down, and cases continued to increase rapidly—yet, as the Israeli part of the research team testified, this period *felt* less dramatic. While cases were increasing, these were not the apocalyptic rates predicted by public health authorities. Israel—a nation accustomed to public crises (Cohen, 2002; Peri, 2007)—seemed to quickly become habituated to the Coronavirus as part of daily life.

## Twitter Use in Times of Global Crisis—Evidence from the Field

Before moving to the experiences of our respondents—salient Twitter users in Jerusalem—some context is needed on crisis communication in Israel. In its 72 years, Israel has known a range of public crises, mostly in the form of armed conflict. Cohen (2002) describes Israel as a “crisis-ridden democracy,” whose media structure is accustomed to covering national crises, often through a live, “non-stop, open-ended broadcasting mode” of “regurgitating disaster” (Liebes, 1998).

National crises are also reflected in Israelis’ use of social media—as studied extensively in the context of the Israel-Gaza conflict of 2014. In the context of this crisis, Malka et al. (2015) found that Israelis used WhatsApp to consume information and interact socially. John and Gal (2018) found that during this conflict, Facebook users pruned political content and contacts from their feed in an attempt to exert control over their “personal public sphere.” While Facebook and WhatsApp are the dominant mainstream social media, Twitter is considered a niche platform in Israel, used particularly by “elite” users such as journalists, politicians, and public influencers. As such, it plays an important role during national crises (Tenenboim, 2017).

Our Jerusalemite Twitter users—many of whom belong to this ‘information elite’—are thus no strangers to public crises. How did they respond to the emerging pandemic? We answer this via a mixed-methods analysis of our two survey waves. Given the literature on social media use during crises in the Israeli context, we would expect a heightened use of social media for news consumption and social coping in Wave 1, with some evidence of “normalization” in Wave 2—possibly reflecting Israelis’ quick habituation to crisis.

We begin with a quantitative analysis. Before each survey wave, respondents filled out measures about intensity of, and motivations for, general Twitter usage (adapted from Alhabash & Ma, 2017). Our added post-survey “Coronavirus questionnaire” included these measures again, but asked respondents to think specifically about their Twitter use during the previous 10 days (Wave 1, *N* = 34; Wave 2, *N* = 25; Gender: 16 women, 42 men; Age: *M* = 38.17 years, *SD* = 12.31 years).

As Table 1 shows, the two survey waves were very similar in terms of intensity and motivations of Twitter usage. In line with our sample of frequent users of Twitter, intensity of Twitter usage was already high pre-survey, for both waves. For Wave 1 users, one item for *intensity* of usage increased significantly post-survey: “I would be disappointed if Twitter shut down.” In terms of *motivations* for Twitter usage, one item increased significantly, for both waves: “I use Twitter because it helps me pass the time.” Borderline significance was found for the item “I use Twitter to receive information” for Wave 2 only, with agreement *decreasing*. This may confirm a sense of “Coronavirus habituation” (or perhaps, Coronavirus information-overload) for Wave 2 respondents.

**Table 1.** Intensity and motivations for Twitter Usage across the two waves.

	Wave 1 (*n* = 34)			Wave 2 (*n* = 25)			Whole sample (*n* = 59)		
	Pre-survey (general Twitter use) Mean (*SD*)	Post-survey (Twitter during Coronavirus) Mean (*SD*)	*p* (Wilcoxon signed-rank test)	Pre-survey (general Twitter use) Mean (*SD*)	Post-survey (Twitter during Coronavirus) Mean (*SD*)	*p* (Wilcoxon signed-rank test)	Pre-survey (general Twitter use) Mean (*SD*)	Post-survey (Twitter during Coronavirus) Mean (*SD*)	*p* (Wilcoxon signed-rank test)
Intensity of Twitter usage									
Twitter is (was) an important part of my daily activities.	5.71 (1.64)	5.65 (1.48)	1	5.72 (1.57)	5.24 (1.64)	0.19	5.71 (1.6)	5.48 (1.55)	0.31
I feel (felt) out of touch when I have not (had not) logged onto Twitter.	4.79 (1.9)	4.85 (1.81)	0.92	5.12 (1.79)	4.6 (1.56)	0.33	4.93 (1.85)	4.75 (1.7)	0.56
I would be (would have been) disappointed if Twitter (had) shut down.	5.35 (1.89)	6.03 (1.19)	0.04*	5.52 (1.64)	5.72 (1.51)	0.48	5.42 (1.77)	5.9 (1.34)	0.03*
Motivations for Twitter Usage									
I use (used) Twitter to share information.	5.88 (1.27)	5.85 (1.46)	0.84	5.42 (1.77)	5.32 (1.7)	0.81	5.69 (1.5)	5.63 (1.58)	0.72
I use (used) Twitter to record what I do (did) in my life.	3 (1.7)	3.12 (1.65)	0.53	2.8 (1.89)	2.84 (2.21)	0.97	2.91 (1.77)	3 (1.89)	0.54
I use (used) Twitter because it entertains (entertained) me.	4.38 (1.98)	4.47 (1.88)	0.26	4.56 (1.85)	4.48 (2)	0.61	4.46 (1.91)	4.48 (1.92)	0.59
I use (used) Twitter to connect to people.	4.47 (1.83)	4.65 (1.59)	0.48	4.36 (1.85)	4.6 (1.53)	0.4	4.42 (1.82)	4.63 (1.55)	0.29
I use (used) Twitter because it helps (helped) me pass the time.	3.47 (2)	4.74 (2.04)	**<** 0.001***	3.64 (2.14)	4.76 (1.81)	0.03*	3.54 (2.05)	4.75 (1.93)	**<** 0.001***
I use (used) Twitter to tell others about my personality.	3.21 (1.83)	2.88 (1.63)	0.33	3.36 (2.23)	3.2 (2.14)	0.55	3.28 (2)	3.02 (1.85)	0.22
I use (used) Twitter to receive information	6.18 (1.49)	6.21 (1.18)	0.71	6.16 (1.41)	5.72 (1.75)	0.05*	6.17 (1.44)	6 (1.45)	0

Pre-survey, respondents were surveyed about their general Twitter usage, unrelated to the Coronavirus crisis (*General Twitter Use*). At the end of the survey period, they were asked to reflect again on these items, this time relating to the last 10 days, during the height of the Coronavirus crisis (*Twitter during Coronavirus*). Instructions were “How strongly do you agree with the following statements?” (*General Twitter Use*) or “When you’re thinking specifically about the past 10 days, how strongly do you agree with the following statements?” (*Twitter during Coronavirus*). Items were rated on a 7-point Likert-type scale with the extremes labeled “Do not agree at all” versus “Strongly agree.” Respondents were surveyed in two waves. The first wave (*n* = 34) was given the COVID-19-related questionnaire on March 19, 2020 and the second wave (*n* = 25) on April 2, 2020.

* *p*<.05. ***p<.001.

While most aggregate measures were very similar pre- and post-survey, some evidence suggests the existence of underlying changes. Mapping movements between categories from the pre-survey to the post-survey shows for many of the items *movements from roughly equal numbers, in opposite directions*. The most illustrative example was the item “I feel out of touch when I haven’t logged onto Twitter.” Although this item did not change significantly from pre-survey to post-survey, only ten participants agreed exactly the same with this statement in both questionnaires; 25 agreed less and 24 agreed more in the post-survey questionnaire. Beyond this item, most movements are small- or medium-sized (changes of 1–2 points on a 7-point Likert-type scale), suggesting that most people adjusted their behavior, but did not completely overhaul it during the crisis. However, there are *some* people whose answers shifted much more dramatically.

These data imply that the Coronavirus shaped different respondents’ Twitter use in very differential, even opposing ways. To further investigate this variance, we employed a qualitative thematic analysis of responses to the question “how do you feel the Coronavirus situation shaped your Tweeting habits over the past 10 days?.”

Our analysis points at three emerging themes: Information goals, social connections, and use habits.

*Information goals* was the most salient theme, reflecting use of Twitter to stay up-to-date on the unfolding Coronavirus situation. One of the unique socio-technical affordances of Twitter was the ability to find in-depth analysis in a quickly changing reality:

Twitter was useful in searching and interpreting nearly-live data about the origin and spread of the disease. [. . . ] This was easier done on Twitter than on traditional news media. (Male, 33, Wave 1)

It enabled me to follow the literature, the science, the scientific breakthroughs and those that are controversial, and to publish a daily graph with the number of cases in Israel vis-à-vis the expected exponential growth. (Male, 49, Wave 2)

While users in both waves described using Twitter for Coronavirus-related information, an aspect that was unique to Wave 1 was Coronavirus-related content replacing other topics:

Tweeted less about tech/work, more about Corona. (Male, 34, Wave 1)

It has meant a focus on only 1 topic. . . (Female, 48, Wave 1)

A second pertinent theme was using Twitter for *maintaining social connections*. This function was particularly pronounced vis-à-vis stay-at-home orders:

Because you’re stuck at home all day it’s the easiest way to check up what’s going on with everyone. (Male, 23, Wave 2)

A small number of respondents specifically mentioned dealing with psychological difficulties, such as anxiety, stress, and loneliness. These were connected to both increased and decreased use of Twitter:

The anxiety and preoccupation with local, immediate issues makes it harder to focus on Twitter. (Male, 68, Wave 1)

I felt less lonely [when using Twitter during the Coronavirus period]. (Female, 46, Wave 2)

Findings in the final theme—*use habits*—provide further substance to the suggestion that the Coronavirus situation affected different respondents’ Twitter usage in differential ways, even reflecting oppositional movement. Especially in Wave 1, we saw reports of increased use:

I used Twitter much more—both as a source of information about news pertaining to the Coronavirus and also as a community. (Male, 37, Wave 1)

I log on much more. Average of 4-6 log-ins a day, now I log in about every hour. Sometimes more. (Female, 35, Wave 1)

While for fewer participants, closure of schools and the presence of family members led to decreased available time:

Logistics and having kids at home all of the time changes my social media habits. [. . . ] I did not participate on Twitter in as many conversations or with regular online activities as much as usual by far. (Male, 65, Wave 2)

In both waves, however, a sizable portion of users reported no noticeable change in their social media use (or, sometimes, in their lives), seeing the Coronavirus as “an event like any other in terms of my Twitter use” (Male, 42, Wave 1).

Taken together, our findings depict the Coronavirus as a unique type of crisis in terms of shaping social media use. For most participants, it was marked by home isolation, explaining the increased use of Twitter as a way to pass time—a motivation rather uncommon in “traditional” crises such as natural disasters (Takahashi et al., 2015). Beyond that, we see some evidence for differential impacts on different participants, depending on how the pandemic affected their life contexts. Future research on COVID-19 and media should take into account possible factors shaping such “differential effects,” for example, employment status (did the respondent switch to working from home? Did they become unemployed?), parental status (especially given school closures), or close relations to people sick with COVID-19. At the same time, some lessons from existing crisis communication research may also apply for this unique crisis (e.g., in Israel, relatively quick habituation). Methodologically, our study points at the value of combining standardized measures with open-ended approaches, particularly for understanding novel phenomena like COVID-19. Finally, our project exemplifies how researchers have to suddenly embrace entirely new conditions of social reality in order not to let this (and other) crises go to waste.

## Notes

1. This research is funded by the DFG (Deutsche Forschungsgemeinschaft/German Research Foundation)—project number 290045248—SFB 1265.

2. We recruited participants out of a pool of individual users in each city who posted frequently on Twitter.

3. Our early hunch that the Jerusalem data would be significantly shaped by COVID-19 related tweets, rendering a comparison to the Berlin data irrelevant, was supported when the data came in. To illustrate, we examined both datasets for use of five Coronavirus-related keywords (Corona, Covid, Wuhan, Virus, and China) in Tweet topics. These topical keywords were used to describe only 1.4% of Tweet instances in the Berlin dataset (13/927) versus 26.2% (166/633) in the Jerusalem dataset.
